# Palliative Care Services for Indian Migrants in Australia: Experiences of the Family of Terminally Ill Patients

**DOI:** 10.4103/0973-1075.53589

**Published:** 2009

**Authors:** Sujatha Shanmugasundaram, Margaret O'Connor

**Affiliations:** School of Nursing and Midwifery, Monash University, Peninsula Campus, Frankston, Victoria, Australia - 3199; 1 Vivian Bullwinkel Chair in Nursing, Palliative Care, School of Nursing and Midwifery, Monash University, Peninsula Campus, Frankston, Victoria, Australia - 3199

**Keywords:** Cultural issues, Indian migrants, Palliative care, Terminal illness

## Abstract

**Background:**

The way that health care systems in developing countries like India care for dying patients, has an impact on the expectations of such care for those who migrate to other countries faces. At the end of life, cultural issues may impact on the quality of life remaining and for that reason, it is important that particular cultural practices are understood. This paper describes a study that investigated the cultural issues of access to palliative care services for Indian migrants in Australia.

**Purpose of the Study:**

To investigate the experiences of the family members of terminally ill Indian migrants in Victoria, Australia.

**Objective of the Study:**

To explore the issues related to accessing palliative care services for Indian migrants; to identify the effectiveness of palliative care in supporting the patient and family and to recommend strategies for improving this care.

**Materials and Methods:**

A qualitative descriptive design was utilized. Up to 6 family members were selected for in-depth interviews in understanding cultural issues related to the palliative care services for a family member.

**Results:**

Analysis of the interviews revealed that families of Indian patients experience difficulties whilst receiving palliative care services, which fell into three main categories: Indian support systems, cultural issues, and caring experiences. Although each of these issues had a direct influence on the experience of terminal care that their family member received, cultural issues and support systems also influenced the caring experiences.

**Conclusion:**

Despite the successful implementation of palliative care services across Australia, there are still problems in accessing and receiving the services among minority and disadvantaged groups like various cultural groups.

## INTRODUCTION

The current global health care system has a massive challenge in providing need-based care to people, particularly as they near the end of life. In recent decades, life expectancy has been increasing in most developed countries, with more and more people living beyond 65 years of age.[1] As part of the population ageing, the distribution of diseases people suffer and die from is also changing. Increasingly, people die at older ages following illnesses related to serious chronic diseases, which cause a wide range of physical, psychological, and social problems.[2] Health care systems must be able to meet the needs of these people by reducing suffering and supporting people of all ages to have optimum health and maintain their quality of life as long as possible. Within these frameworks, palliative care has been globally recognized as a vital component in the health care system[3] and palliative care is fast becoming an area for delivering health care for people with life-limiting illnesses.

“Palliative care provides relief from pain and other distressing symptoms, affirms life and regards dying as a normal process, and intends neither to hasten nor to prolong death. Palliative care integrates the psychological and spiritual aspects of patient care and offers a support system to help patients live as actively as possible until death. It also offers a support system to help the family cope during the patient's illness and in their own bereavements.”[4] Using a team approach, palliative care addresses the needs of patients and their families, including bereavement counseling if necessary. Changing patterns of the care of terminally ill people and preference for death at home means there is often a heavy reliance on family caregivers to provide the majority of the care needed.[5]

Palliative care enhances quality of life and may positively influence the course of the illness. Palliative care is applicable early in the course of the illness with other therapies that are intended to prolong life, such as chemotherapy or radiation therapy, and includes those investigations needed to better understand and manage distressing clinical complications.[6] Palliative care should be offered as needs develop and before they become unmanageable; it should be an integral part of the care in any setting and this is a major challenge both clinically and culturally, which requires health care providers to work together to meet the needs of some of the most vulnerable patients, their families, and carers.

It is against this environment that the researcher engaged in a dialogue with Indian families to find out what their experiences had been during the illness trajectory. The objectives of the study were to explore the issues related to accessing palliative care services for Indian migrants; identify the effectiveness of palliative care in supporting the patient and family; and make recommendations for improving care for the family members of terminally ill Indian migrants.

## LITERATURE REVIEW

### Palliative care services

The Hospice Information Service website[7] estimated that in 2005 there were hospice/palliative care services, either existing or under development, in about 100 countries around the world with between 7000 and 8000 palliative care initiatives including community based teams, inpatient units, and day care centers.[7] The distribution of these services is however, heavily weighted toward western countries. Only about 6% of all palliative care services are located in Asia and Africa, the regions where the majority of the world's population lives and dies.[8]

The data on developing countries suggests that even in those countries where palliative care services exist, the coverage of many of the programs is limited and palliative care is not on the health agenda of their governments as a public health problem. This limits the ability of palliative care services to impact on the populations that most need them.[9–13]

The knowledge already exists about how to improve the quality of life of those who are dying particularly in regard to pain relief, but it is not widely practiced.[14] As noted above, this is evident in developing countries and is partly attributable to the usual reasons for poor health care of any description including poverty and lack of basic needs (food, clothes, and clean water), lack of resources and inadequate health system infrastructure. There are, however, three specific barriers to palliative care implementation that are generally accepted as the major impediments: lack of government commitment, opioid availability, and limited education.[15,16]

The applications of palliative care to a broader range of conditions are not straight forward. A major issue is the identification of patients with a terminal illness other than cancer who are likely to have a significantly reduced life expectancy because of their condition. The demand for palliative care services is increasing and this trend is likely to continue given the ageing of the population, an increasing prevalence of advanced cancer, the emergence of new incurable diseases such as AIDS, and a community more informed about issues related to death and dying.[17,18]

Firth[19] suggests that access to palliative care services by minority ethnic groups is lower than their proportion in the local community would suggest. There are many factors contributing to this problem, including ignorance about services, lack of accessible and appropriate information, and little confidence in the ability of services to understand and meet their needs. They may perceive hospices as places where white people die, be anxious about appropriate religious and cultural care, and concerned about communication.

The other common reason for not accessing service is lack of referral. Evidence suggests that there are differences between the people referred to various types of palliative care services. Kristjanson *et al.* reported on terminally ill people referred to three different types of palliative care service and showed that those referred to inpatient services were older, while the clients of the home care service were more likely to have a primary caregiver. Another study also found that patients selected for inpatient palliative care were older, highly dependent because of difficulties with activities of daily living and had a short life expectancy.[20]

As noted earlier, there are identifiable groups in society who are missing out on palliative care services.[21] The reasons may vary between different cultural groups, but a major problem faced by health care professionals and those from different cultural and non-English speaking backgrounds is the difficulties of communication. Literature reports that there is poor access to palliative care services among minority ethnic groups not only in Australia but also in the United States and United Kingdom.[22] The common barriers identified were poor communication, lack of information provision in appropriate languages and lack of local information networks for these particular groups of people.[23]

### Care giver experiences of terminally ill

Although cure is not always possible, human caring is the critical element in achieving a peaceful and dignified death. The care-giving experience can be a shared responsibility of the members of the community who support the dying and their families in meaningful ways. People may die in the comfort of their own home, close to their loved ones, with death as much a part of the collective consciousness as birth.[24]

One of the fundamental principles of palliative care is that the patient and family together are the unit of care.[25] There is encouragement to shift end of life care from hospital settings where health care professionals are in-charge, to the home care setting where the family members are in-charge of the care.[26]

Lynn[27] and Duhamel and Dupies[28] note that suggested domains for measuring quality at the end of life should include patient and family satisfaction and family burden including the financial and emotional burden. Patient and family satisfaction should include the patient's peace of mind, the family's perception of the patient's care and comfort, the decision making process, the care received, both by the patient and the family, and the extent to which opportunities were provided to complete life in a meaningful way. The time spent by patient and family should be treasured and not simply tolerable.

Despite the successful implementation of high-level palliative care services across Australia, most terminally ill patients, regardless of their ethnic background, prefer to die at home and need to be taken care of by family members and friends. The family members often experience enormous stress whilst taking care of terminally ill patients.[29] Studies of the emotional consequences of care giving reveal that relatives of cancer patients may experience as many psychological problems as that of the patient. These include anxiety, depression, low self-esteem, feeling of isolation, mental fatigue, guilt, and grief.[21] Family care giving does have a negative impact on the family's quality of life. Almost a third of Australian family caregivers reported confronting significant anxiety and 12% experienced significant depression. In most circumstances caring for dying relatives at home is a matter of honor and integrity for many families of different cultures; failure to do so creates stigma and loss of face.[30]

For many South East Asian families decision making is patriarchal. Male power roles are reinforced if the women do not speak English and are dependent on their husbands or sons to translate. This creates problems for the nursing staff in communicating with female patients.[31] There are real dilemmas in respecting their situation in their particular context.[32] Tensions can arise when an elder needs care from a female relative who has quite different expectations. One's culture has profound religious implications and implications for moral identity in fulfilling certain obligations set into the context of the wider community.[33] Thus, Indians live with extended families to provide additional support for the dying patient and their immediate family members. As families struggle to be caregivers of the dying, they must also struggle with their impending loss, their changing roles and relationships, watching a loved one suffer both physically and emotionally, and dealing with their own concerns about their ability to be a caregiver.[5]

Despite these difficulties, a study conducted on satisfaction of life at the end of life among bereaved carers found that as caregivers their satisfaction was high; 87% of participants gave community nurses a rating of excellent and 77% of participants gave general practitioners rating of good or excellent. Another study conducted by Lecourturier *et al*. on bereaved carers showed that specialist services received by the family members reports of higher satisfaction levels.

## MATERIALS AND METHODS

A descriptive research design was used, appropriate when little is known about a research area and the objectives are to elicit detailed descriptions of the phenomena in question.[35] No predefined conceptual frame work was used to direct this study and this is consistent with qualitative descriptive studies.[35] Rather, the background literature provided a guiding orientation to the development of the research questions, methodological decisions and construction of the interview guide. The intent of the study was to allow the participants to generate descriptions of their experiences, rather than predetermined categories of responses.

### Participants

On receiving ethical approval from the researcher's academic institution and clinical sites of research, six family members were recruited to be interviewed. Interviews were conducted in English. All were the primary caregivers of the patients, ranging in age from 47 to 68 years. Four were female, two being wives and two the daughter-in-law of the patients; the males were all husbands of the patients. Five patients had a diagnosis of advanced cancer and one had had a stroke. The patient ages ranged from 51 to 95 years of age.

Of the 6 participants four were Hindus, one was Sikh, and one was Christian and all spoke English. However, the first language was as follows: Tamil (n = 2), Punjabi, (n = 2), Malayalam (n = 1), and Hindi (n = 1). The participants had lived in Australia from 12 to 25 years. Of the six participants, four of their family members were receiving an inpatient service, one was receiving home care and the other was receiving outpatient care. The caregivers had been caring from 5 months to 8 years. All patients had died except one (patient-6) at the time of data collection.

The study was undertaken in Melbourne because much of the Indian population live in South East Suburbs of Melbourne. A convenience sample of family members of six Indian patients who had required, were receiving palliative care services were selected for the study. Other inclusion criteria were that the family member was required to be over 18 years of age, could understand, speak and read English and, in the event that their family member had died, the death had occurred less than 12 months ago.

### The recruitment process

Three strategies were used to recruit participants. Nurses from the inpatient and community palliative care services involved in the study were asked to identify potential participants who met the above criteria and offered a letter of invitation. If willing to participate in the study, they then contacted the researcher directly.

A second strategy sought the involvement of local general practitioners, to forward a letter of invitation to the family of current or past patients of Indian descent that required terminal care. The final strategy was to use a snowball sampling technique, asking families who agreed to participate if they knew other Indian families who might be eligible for the study and if so, to pass on a copy of the letter of invitation to them.

### Ethical considerations

Permission to conduct the study was obtained from the university's ethics committee and as well as the in-patient and home based setting where the research was carried out. Prior to the interview, a participant information sheet and consent form was provided, describing the purpose of the study, the inclusion criteria, and the commitment involved. Before the interview, each participant was asked to complete a consent form that outlined the purpose of the study, the format of the interview, and the maintenance of his or her confidentiality. Participants were also informed that their participation was voluntary and they could withdraw from the study at any time. With permission, all interviews were recorded and the audiotapes were transcribed by the researcher into the computer.

### Data collection

The researcher firstly collected the background demographic details like age, gender, religion, relationship to the patient, language, and length of stay in Australia. Then face-to-face interviews were conducted, comprising six open ended questions seeking understanding about the caring experiences of these family members.

The interviews were conducted in participant's home at a convenient date and time. If the participant became distressed or felt uncomfortable with the interview, the interview was stopped immediately. All the interviews were conducted in English, as all were fluent in English. Each interview lasted from 35 minutes to 1 hour and was audiotaped with their consent. Probing was used to ensure the credibility of the data and reduced the risk of socially desirable answers.[36] The researcher acknowledged her preconceived ideas on the study topic and adopted an open attitude of learning from the personal experiences of the family members.[37] Before starting the interview the researcher made sure privacy was provided to the participants and confidentiality assured. Following the completion of each face-to-face interview, the researcher completed field notes, meant to summarize the contextual characteristics of the interview, which may have been lost on reading the transcript at a later date. These notes were then used to inform the analysis of the data.

### Data analysis

Patton's method of phenomenological analysis was used to analyze this data.[38] This method of analysis is that it “seeks to grasp and elucidate meaning, structure and essence of the lived experience of a phenomenon for a person or a group of people.”[38] It was this outcome with regard to the caring experiences described by family members that was sought. A form of analysis involving Glasser and Strauss’ method of constant comparison of themes was employed to elicit common themes and patterns.[39] The researcher was confident that saturation had been reached when replication of themes was found in the data.

## RESULTS

The analysis identified three major findings: Indian support systems, cultural issues, and caring experiences which are now discussed.

### Support systems for Indian people

Many of the respondents faced significant cultural challenges when they required health care services for their loved one. They described a lack of sensitivity to many cultural issues and dissatisfaction with the care provided, causing one interviewee to suggest that:

“…” need an Indian nursing home in Australia, because my mother in law does not like western food and she does not take meat “…” (Case-3)

Generally, the participants seemed unaware of the breadth of health care systems available in Australia. Most of the caregivers did not know about the availability of palliative care services and the assistance they could receive. Many caregivers said they came to know about palliative care when their relative was admitted to hospital and was subsequently referred to a palliative care service.

We do not know what they exactly do in the palliative care but, whatever problems the patient has, the doctors come and attend the patient immediately and resolve the problems like pain, breathing difficulty and urinary infection etc. (Case-4)

### Cultural issues

Food seemed to be a major problem for most of relatives of the participants. Indians eat their traditional foods even in Australia, which imposes significant stress on family members when a person becomes ill. Even if being cared for in an inpatient setting, the relatives were still preparing food and feeding the person, because of the unavailability of culturally appropriate foods.

#### One caregiver expressed

“My wife is a vegetarian and she takes Indian breakfast in the morning at 7.30 or 8 am, so it was hard for us to prepare from the home and give it to her in time.” (Case-1)

Most of the participants described food as medicine for their relative. Sometimes food was prepared with fresh green leafy vegetables and herbs available from the garden. The Hindu participants described their preference to eat vegetables and fruits as fresh as possible. More so, because they worship the cow as a god, they do not eat beef and are more likely to be vegetarian; most would eat a vegetable curry daily. In most food preparation, participants described their usage of ginger and garlic, because ginger helps in digestion and garlic reduces the cholesterol level in the blood.

#### Other caregiver quoted that

“My husband does not like porridge or bread for breakfast, so I had to prepare Indian dishes and give him. At least then he will be happy you know and I will be satisfied I have done something good for him.” (Case-2)

#### Another caregiver explained

We eat only vegetarian and we are Brahmin caste. But, they give her beef, pork, chicken and lamb… She never likes that and she felt it is not the right food for her… because she is wasting them each time. She takes eggs in the nursing home which she is not supposed to. (Case-4)

As with other religions of the Indian subcontinent, Hindus have particular requirements for modesty and need total privacy for bed baths, examinations and almost any procedures as well as requiring treatments to be given by health care workers of the same sex. Physical cleansing is associated with spiritual cleansing; hence, it is important to the Hindu to wash before they pray. As with most people of Asian origins, there will be the need for comprehensive washing after their use of the toilet, so a container of water should be available.[19] Physical purity is valued, whereby Hindus try to bathe daily in running water, not a bath and require help to do this when they are ill, particularly if they are older. They also prefer to bathe before saying their prayers. Hindus believe that bathing renders one both physically and spiritually clean, so that the desire to bathe can be very strong amongst the terminally ill and they must be assured that help is available.[19]

Family members are likely to be present in large numbers as death nears. Chanting and prayer incense and various rituals are part of the process. After death, health professionals should touch the body as little as possible and ideally, the family should be the only ones to touch it. A family member of the same sex as the deceased should clean the body. After being cleaned, the body is wrapped in a red cloth. The preference is for cremation and ideally, the ashes scattered in the River Ganges. The men and boys of the family shave their hair as a symbol of mourning for the dead. The mourning family wear all white and seek to have a Brahman at the funeral to perform blessing.[19]

One caregiver described her disappointment at the lack of communication from staff as her mother-in-law was dying:

When my mother in law dying staff did not inform us, so we could not go in time, talk to her and spend time with her at the last stage of her life. It is our custom that we have to give her a sip of water when she is dying. (Case-4)

Most Indians in Australia are Hindus, following strict religious rules particularly older people. The older people observe Holy days (Fridays) and the need for ritual fasting.

When my mother in-law was in the nursing home, she could not practice this ritual as she had to take the medicines in the right time. (Case-3)

In relation to prayer, one caregiver expressed their frustration at the lack of acknowledgement of what was needed. In the institutional environment, it seemed difficult to find the quiet space:

There is no separate room for the prayer and there is no privacy… Because the surroundings are noisy, I cannot concentrate on the prayer. So, I just pray at home give her the offerings when I go and visit her in the nursing home… (Case-3)

While family members described their feelings that no medication or therapy could cure their loved one, they still sought care that would help them to suffer less and have a peaceful and good death.

I know my wife is diagnosed as cancer, but luckily she is in early stage of disease. I hope the doctors will cure her illness. I am mentally prepared for anything now (Case-6)

### Care-giving experiences

The level of the burden and the suffering that the caregivers felt was evident in the interviews and thought that it may be greater than that the patient's suffering, especially in the last few weeks of life. Nevertheless, the family members described that it would be difficult to give up caring for their loved one, because they saw it as their last chance to do something for them. In this light, some participants described that they had refused the offer of respite care, at times to the detriment to their own health.

Some of the caregivers described their lack of knowledge about palliative care services, lack of resources, and lack of knowledge about what to expect from the health services. This lack of information also caused them additional burden and mental stress.

#### One caregiver noted

When I heard my husband had a brain cancer, I was shocked. As the disease progressed I went into depression and I had to go for treatment with mental health doctor. (Case-2)

Most of the participants described their care-giving role as ‘hard work’; they were physically tired and additionally burdened:

It's a hard work. Because my husband is aged and I cannot toilet him or shower him, more so, he is fragile and he had a number of falls. For the past weeks he was going down and was vomiting. I just cannot do that and really it is a hard work for me. (Case-2, 55 years old female caring her husband)

Some other participants described the mental stress and strain of their care-giving role:

I have no words to say. It is an experience. It was so painful for me. I feel so much under pressure you know, I have sleepless nights, no one to support me and no one is there to help me, so its lot of mental stress for me especially at the last stage of his illness. I have no time to spend with children. (Case-5, 47 years old female caring her husband)

Most of the caregivers had some idea of the meaning of palliative care. For them however, they believed that if their loved one were transferred into palliative care, he/she would die soon.

About palliative care what I know is, it is the care given to the people with terminal illness, and maintaining and keeping quality of life. That's all I know (Case-4).

## DISCUSSION

The primary aim of this study was to explore the issues to access the palliative care services in Australia. The participants described their different needs and of their ill family member when faced with the complications of living with a terminal illness. They saw themselves as an integral part in providing information about their loved one. However, the literature shows that little is known about Indian family members experiences of being caregivers, as well as their expectations of the palliative care services; hence the importance of this small study.[40]

Although family members visited their loved one in the nursing home or hospital daily or on alternate days and spent time with them, the participants in this study did not feel they received sufficient information about their care. They expected the information regarding their loved one to be given in advance so that they could plan their activities accordingly.

Surprisingly in this study, even though the participants were all well-educated and working as professionals they felt their lack of knowledge on medical terminologies and medical knowledge, caused them unnecessary stress. This experience is echoed in a national survey of written information provided to patients and family members by the palliative care units in the United Kingdom.[22] This study too, found that many patients and families may be unable to gather appropriate written information about palliative care.

The second aim of this study was to identify the effectiveness of palliative care in supporting patients and families. The concept of palliative care is rather complex, but services seek to support not only the patients but also the family members and caregivers.[46] The participants described difficulties in achieving culturally appropriate care as well as in their communication between the health care professionals. Support for the families and other caregivers reduce stress levels and prevent depression by providing adequate information regarding the patient's prognosis in right time.[41] This study highlights that further work is needed to explore the priorities of caregivers and the particular supports required from the services. Family members could be more integrated into the daily routine of the organization for Indian patients, to assist in ensuring the care is individually culturally appropriate. Providing holistic care to both patients and family members in palliative care will achieve more sensitive care at the end of life.[47]

The final aim of this study was to recommend strategies for improving care for family members. Palliative care in Australia has still a long way to go in areas such as improving services for both indigenous and ethnic communities,[42,43] increasing palliative care within aged care facilities, and accumulating an evidence base for palliative care. And while Palliative Care Australia has developed multicultural guidelines for various minority ethnic groups regarding palliative care practices,[44] what is required is more in-depth understanding of particular cultures, in this instance the Indian culture, pertaining to death and dying. The detail of these guidelines will need, for example, to be inclusive of all the languages spoken by Indian people in Australia.

The family members involved in this study expected that their loved one would receive a high level of care even though they were in the end stage of illness. The findings suggest that caregivers felt inadequate and insecure and were socially isolated in their caring role. Attention needs to be given to the support of caregivers of different cultural background. They need to be invited to participate in caregiver support programs and encouraged to participate in bereavement support services after their loved one has died.

There is evidence to suggest that most countries still struggle with a general lack of knowledge and education about palliative care, as well as inadequate death education for health care professionals, patients, and caregivers educational strategies are a possible mechanism to increase the comfort levels of all health care professionals, with their role in giving information earlier and assisting families in talking with dying patients about physical, psychological, spiritual, cultural, and religious aspects of care.[43]

Arnold, suggests that health professionals working in palliative care need “to have a more sophisticated understanding of what is meant by culturally sensitive practice.”[45] Therefore, there is a need for further research in the non-English speaking groups to provide culturally appropriate care in the palliative care settings.

## CONCLUSION

Although palliative care is a complex concept, the opinions of family members involved in this study highlights important issues in relation to the care-giving role for their loved one as well as their perceptions of the care received. The results emphasize the importance of family in assessing and improving culturally appropriate care in a palliative care context in which the family, rather than just the individual, is the focus of care.[48] According to these family member perceptions, it was important for the patient to live a normal, enjoyable life in their own cultural environment, to maintain quality of life during the illness and to be cared for according to the goals of palliative care. This is a challenge not only for palliative care teams but also for organizations, to ensure that it is the individual and family who remain the focus of care.
